# The potential habitat of desert locusts is contracting: predictions under climate change scenarios

**DOI:** 10.7717/peerj.12311

**Published:** 2021-10-26

**Authors:** Jingyun Guan, Moyan Li, Xifeng Ju, Jun Lin, Jianguo Wu, Jianghua Zheng

**Affiliations:** 1 College of Resources & Environment Science, Xinjiang University, Urumqi, Xinjiang, China; 2 Key Laboratory for Oasis Ecology, Xinjiang University, Urumqi, Xinjiang, China; 3 College of Tourism, Xinjiang University of Finance & Economics, Urumqi, Xinjiang, China; 4 Locust and Rodent Control Headquarters of Xinjiang, Urumqi, Xinjiang, China; 5 Institute of Arid Ecology and Environment, Xinjiang University, Urumqi, Xinjiang, China

**Keywords:** Potential habitat, Shared socioeconomic pathways, Climate change, Maximum entropy, Desert locust

## Abstract

Desert locusts are notorious for their widespread distribution and strong destructive power. Their influence extends from the vast arid and semiarid regions of western Africa to northwestern India. Large-scale locust outbreaks can have devastating consequences for food security, and their social impact may be long-lasting. Climate change has increased the uncertainty of desert locust outbreaks, and predicting suitable habitats for this species under climate change scenarios will help humans deal with the potential threat of locust outbreaks. By comprehensively considering climate, soil, and terrain variables, the maximum entropy (MaxEnt) model was used to predict the potential habitats of solitary desert locusts in the 2050s and 2070s under the four shared socioeconomic pathways (SSP126, SSP245, SSP370, and SSP585) in the CMIP6 model. The modeling results show that the average area under the curve (AUC) and true skill statistic (TSS) reached 0.908 ± 0.002 and 0.701, respectively, indicating that the MaxEnt model performed extremely well and provided outstanding prediction results. The prediction results indicate that climate change will have an impact on the distribution of the potential habitat of solitary desert locusts. With the increase in radiative forcing overtime, the suitable areas for desert locusts will continue to contract, especially in the 2070s under the SSP585 scenario, and the moderately and highly suitable areas will decrease by 0.88 × 10^6^ km^2^ and 1.55 × 10^6^ km^2^, respectively. Although the potentially suitable area for desert locusts is contracting, the future threat posed by the desert locust to agricultural production and food security cannot be underestimated, given the combination of maintained breeding areas, frequent extreme weather events, pressure from population growth, and volatile sociopolitical environments. In conclusion, methods such as monitoring and early warning, financial support, regional cooperation, and scientific prevention and control of desert locust plagues should be further implemented.

## Introduction

Desert locust (*Schistocerca gregaria*) is the most widespread and highly mobile destructive pest in the world (Symmons & Cressman, 2001; Latchininsky, 2013; Meynard et al., 2017). Desert locusts usually exhibit two different behavior stages: the solitary stage and the gregarious stage (Uvarov, 1997; Simpson, McCaffery & HÄgele, 1999; Symmons & Cressman, 2001). In the solitary stage, desert locusts usually reproduce at low density throughout the recession areas of North Africa, the Middle East, and northwestern India, covering an area of 16 million km^2^. Once swarms are formed, desert locusts may migrate to a larger area of 29 million km^2^, threatening the livelihoods of people in more than 60 countries or regions (Symmons & Cressman, 2001; Tratalos et al., 2010; CABI, 2021). Desert locusts feed on large amounts of an area’s vegetation, leading to a wholesale destruction of crops, pasture, and fodder (Meynard et al., 2017; Moberly, 2020). Swarming desert locusts pose an unprecedented threat to agriculture-based livelihoods, food security, and national economies in an already fragile region (Brader et al., 2006; Mohammed et al., 2015; Renier et al., 2015; Moberly, 2020). The social impact of locust outbreaks is visible even 20 years after the invasion (Brader et al., 2006). Therefore, predicting the potential distribution of locusts and its changes is particularly important for desert locust monitoring and control, especially in areas that are already very vulnerable.

Climate change, especially global warming, affects the geographical distribution of species on earth (Parmesan & Yohe, 2003; Noce, Caporaso & Santini, 2019; Rodriguez et al., 2020). The life cycle of desert locusts usually needs to go through three stages (eggs, nymphs (hoppers), and adults), and the time it takes to transition from one stage to another is highly dependent on weather patterns (Rainey et al., 1979; Symmons & Cressman, 2001; Cressman & Stefanski, 2016). Because desert locusts usually rely on moist sandy soil for incubation, some studies have pointed out that regional rainfall fluctuations are the main factor affecting the number of locusts (Bennett, 1976; Tratalos et al., 2010). In addition, as the temperature rises, the hatching cycle of the eggs and the growth cycle of the larvae are usually shortened, and the speed of reproduction within populations will increase (Symmons & Cressman, 2001; Cressman & Stefanski, 2016). Unusual weather will change the ecological conditions in arid areas in a short period, which in turn will lead to substantial changes in the population of desert locusts. Abnormal rainfall usually provides warm and moist soil for the breeding of desert locusts, and also promotes the rapid growth of vegetation to meet the high-density gathering of desert locusts for feeding (Tratalos et al., 2010). Previous studies have also emphasized that outbreaks of desert locusts were related to unusual weather, especially abnormal rainfall. For example, the large-scale desert locust invasion in West Africa from 2003 to 2005 is closely related to the abnormal rainfall in the region. From July 2003 to April 2004, the Sahel region and northwest Africa experienced continuous above-average rainfall, which provided ideal breeding and gathering conditions for the desert locust (Ceccato et al., 2007). The torrential rains brought by two unusually powerful tropical cyclones (Mekunu and Pawan) in May 2018 and December 2019 provided a favorable environment for the reproduction, development, and migration of the East African desert locust from early 2019 to 2020 (Salih et al., 2020; Meynard et al., 2020). In addition, the suitability of habitats for desert locust spawning and subsequent reproduction also depends on the presence of certain soil types, sand content, vegetation, and other conditions that are conducive to the reproduction and aggregation of solitary individuals (Despland, Collett & Simpson, 2000; Symmons & Cressman, 2001; Pekel et al., 2009). The spreading potential of desert locusts depends on their climatic niche and on ecological barriers to dispersal, which may shift with climate changes, such as global warming and extreme weather events (Meynard et al., 2020). According to IPCC (2014) reports, Africa has a very high risk of future climate change, and its ability to deal with future climate change is very fragile. Therefore, climate change poses a great challenge to the monitoring and control of desert locusts. It is extremely important to predict suitable areas for desert locusts in the early stage (solitary period) under different climate change scenarios to prevent large-scale epidemics.

Species distribution models (SDMs) are currently the most popular technology for predicting areas suitable for species and can forecast the impact of climate change on the geographic distribution of species (Elith et al., 2011; Merow, Smith & Silander, 2013). Desert locust habitats can be predicted through SDMs (Meynard et al., 2017; Gómez et al., 2020; Kimathi et al., 2020). As a machine learning algorithm, the maximum entropy (MaxEnt) model is widely used in species distribution modeling. It quantifies the relationship between species and their environment based on the existence of species records, thereby generating potential habitat suitability maps and allowing inferences under future climate scenarios (Phillips et al., 2006; Phillips & Dudík, 2008; Elith et al., 2011; Fois et al., 2018; Rodriguez et al., 2020). Compared with other statistical methods, MaxEnt provides higher predictive performance when species-absence data are not available (Elith et al., 2011; Merow, Smith & Silander, 2013).

In recent years, some studies on the prediction of the potential habitat of desert locusts have been carried out based on SDMs. For example, Gómez et al. (2020) and Kimathi et al. (2020) respectively used different SDMs to predict the potential suitable and breeding regions of desert locusts in the current environment. However, neither of them involved research on the impact of future climate change scenarios on the potential habitats of desert locusts. Meynard et al. (2017) predicted shifts in the potential recession range of desert locusts in the 2050s and 2090s, but this study used the A1B and A2 scenarios released by the IPCC in 2007. The Coupled Model Intercomparison Project phase 6 (CMIP6) provides a further improvement to existing models (CMIP5). CMIP6 proposes new development paths that comprehensively consider future emissions and the social economy: shared socioeconomic pathways (SSPs) (O’Neill et al., 2017; Riahi et al., 2017). CMIP6 models usually have finer resolution and improved dynamic processes and are considered the most advanced climate model simulations to study the effects of past, present, and future climate change (Eyring et al., 2016; O’Neill et al., 2017). Given the limited status of the research on future habitat changes of desert locusts output by the CMIP6 model, Saha, Rahman & Alam (2021) used the MaxEnt model and CMIP6 model output to predict the potential global distribution of desert locusts in 2050s and 2070s. However, the study only used one global climate model (BCC-CSM2-MR). Research shows that a single GCM has a large uncertainty in climate simulation, and the average of multiple models can effectively reduce this uncertainty (Fan et al., 2020). In addition, the study only selected climatic factors as environmental variables and has not comprehensively considered environmental factors such as soil sand content and topography that are closely related to the survival of desert locusts.

Based on the comprehensive consideration of climate, soil, and terrain environmental variables, this study predicted the potential habitat of desert locusts under different climate change scenarios at present and in the future through the Maxent model. Designed to solve the following questions: (1) What are the most important environmental factors affecting the geographic distribution of the solitary desert locust? (2) What is the potential geographic distribution of the desert locust during the solitary stage? (3) What will happen to the suitable habitat of the desert locust during the solitary stage in the 21st century under different CMIP6 scenarios? The results will help managers determine the future suitable habitat for desert locusts, formulate guidelines for preventing and controlling the spread of harmful insects, and maintain the balance of ecosystems.

## Materials & methods

### Occurrence data

**Figure 1:**
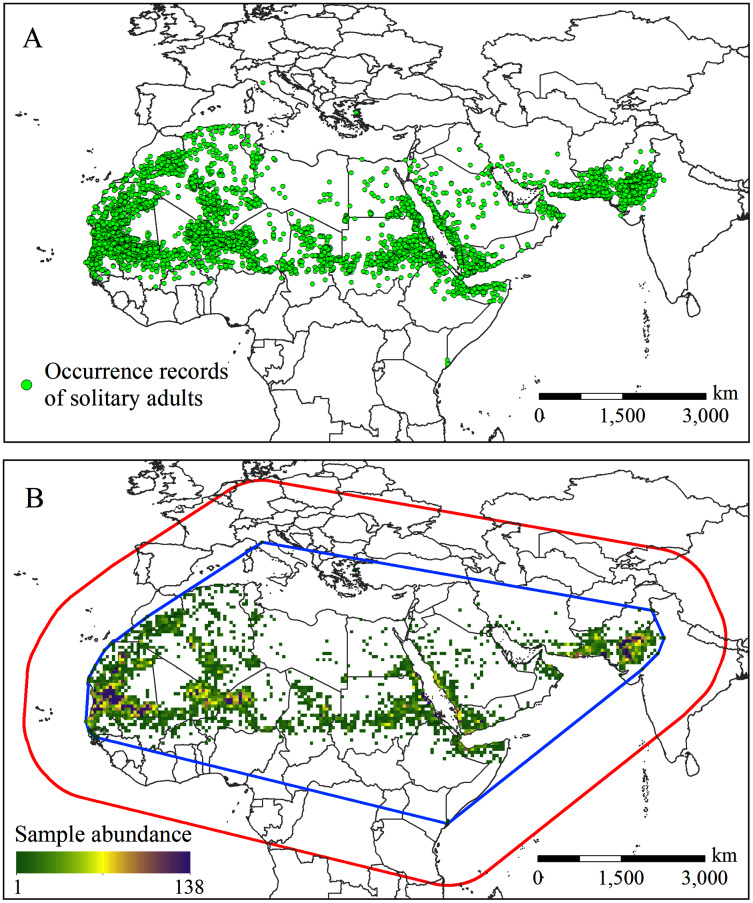
(A) Occurrence records and (B) the spatial abundance of the solitary desert locust samples. The blue outline in (B) is the minimum convex polygon formed around the occurrence records. The red outline represents the SDM background point sampling area, which is formed by a 1,000 km buffer around the minimum convex polygon.

The Food and Agriculture Organization (FAO) of the United Nations has long been devoted to the monitoring and management of desert locusts. In the 1960s, the FAO established a long-term survey database on desert locusts. The data collected include information on the solitary and gregarious stages of the desert locust (Meynard et al., 2017; Gómez et al., 2020; Kimathi et al., 2020). The FAO Locust Hub (https://locust-hub-hqfao.hub.arcgis.com/) currently provides historical records of the desert locust in the solitary period from 1985 to the present. To be as consistent as possible with the current climate data period (1970–2000), we screened records recorded mainly before 2000. In addition, we also cleaned up the occurrence data through the following steps: (1) deleted duplicate and lacking geographic reference records; (2) deleted records of small islands in the Atlantic and Indian Oceans because assigning coarse-resolution coordinates (0.5° × 0.5°) to these records may cause the corresponding climate data to be inaccurate (Li et al., 2014); and (3) deleted records located in the sea but close to the coast at the coarse resolution (0.5° × 0.5°). In the end, 13,970 records were left for subsequent analysis (Fig. 1A).

Because MaxEnt is sensitive to sampling bias (Phillips et al., 2009), to reduce the possible impact of sampling bias on the model output, we used spatial filtering to process the sample points. Spatial filtering is suggested as a measure to mitigate sampling bias and spatial autocorrelation, which can effectively improve the performance of the model, and has been widely used in SDMs modeling. (Boria et al., 2014; Guo et al., 2019; Li et al., 2019; Wang et al., 2019). We put all the occurrence records into a 0.5° × 0.5° grid, assuming that as long as there are one or more occurrence records, a particular grid cell is suitable for the survival of desert locusts. In total, 2,038 grid cells were contained desert locusts occurrence records (Fig. 1B). Finally, those 2,038 grid cells were converted into points based on the pixel centroid, and the latitude and longitude coordinates were recorded for MaxEnt model building.

### Environmental variables

Initially, we collected 23 environmental variables that may affect the spatial distribution of solitary desert locusts (Table S1). Nineteen bioclimatic variables and three topographic variables with a spatial resolution of 10 arc-minutes were downloaded from the WorldClim database (version 2.1, https://www.worldclim.org/), and one soil variable with a spatial resolution of 30 arc-seconds was obtained from the World Soil Database (http://www.fao.org/). Aspect and slope data were generated based on elevation data. We selected the climate data simulated by the eight global climate models (GCMs) provided by the CMIP6 model as the future bioclimatic variables. The eight GCMs were BCC-CSM2-MR, CNRM-CM6-1, CNRM-ESM2-1, CanESM5, IPSL-CM6A-LR, MIROC-ES2 L, MIROC6, and MRI-ESM2-0. Four climate change scenarios (SSP126: +2.6 W·m^−2^, a green development pathway; SSP245: +4.5 W·m^−2^, a middle development pathway; SSP370: +7.0 W·m^−2^, a rocky development pathway; and SSP585: +8.5 W·m^−2^, a high development pathway) considered in two time periods, 2041–2060 (2050s) and 2061–2080 (2070s) (O’Neill et al., 2017; Riahi et al., 2017). For consistency with the spatial resolution of the species occurrence data, all the environmental variables were resampled to 0.5° × 0.5° using bilinear interpolation.

Autocorrelation and multicollinearity between environmental variables may lead to overfitting predictions (Dormann et al., 2013). Spearman correlation and variance inflation factor (VIF) are usually used to assess the multicollinearity of environmental variables (Dormann et al., 2013; Moraitis, Valavanis & Karakassis, 2019). First, based on Spearman’s correlation coefficient and jackknife analysis, the 23 initially selected environmental variables were rescreened. When Spearman’s |r| between a pair of variables was >0.75, we retained the variable that had a higher percentage contribution to the MaxEnt model for subsequent analysis (Wang et al., 2019; Rodriguez et al., 2020). Then, a multicollinearity test was performed on the selected environmental variables with VIF, and the VIF values of all the environmental variables were less than 5. Ultimately, 10 environmental variables were selected for model prediction (Table 1).

**Table 1:** The environmental variables and their VIF values were used for modeling in this study.

Variable	Describe	VIF
Bio1	Annual mean temperature (°C)	3.031
Bio4	Temperature seasonality (standard deviation × 100)	1.916
Bio8	Mean temperature of wettest quarter (°C)	3.190
Bio12	Annual precipitation (mm)	3.018
Bio14	Precipitation of the driest month (mm)	1.687
Bio15	Precipitation seasonality (Coefficient of Variation)	2.308
Bio18	Mean precipitation of the warmest quarter (mm)	2.378
Bio19	Mean precipitation of the coldest quarter (mm)	1.542
Slope	Slope (°)	1.110
T-SAND	Topsoil sand Fraction (%wt.)	1.147

### Modeling process

The MaxEnt model (v.3.4.1; http://www.cs.princeton.edu/~schapire/maxent) was used to build models based on environmental variables and species records (Phillips et al., 2006). The MaxEnt model parameters were set as follows: 75% of the occurrence data were randomly selected for training the model and the remaining 25% were selected for testing (Guo et al., 2019; Liao et al., 2020; Zhang et al., 2020). We set 20 sets of regularization multipliers (0.5–10, with an interval of 0.5), and six different feature combinations (L, LQ, H, LQH, LQHP, LQHPT; where L = linear, Q = quadratic, H = hinge, P = product, T = threshold) to optimize the MaxEnt model (Li et al., 2021). The above process produced a total of 120 parameter combinations. Subsequently, the optimal modeling parameters were selected according to the area under the receiver operating characteristic (ROC) curve (AUC) value. Different sampling ranges of background points will have different effects on the model results. Therefore, the scope of the background points or research area should be carefully selected in the actual application process (Anderson & Raza, 2010; Merow, Smith & Silander, 2013). Generally, the areas that species can access are ideal areas for model development, testing, and comparison (Barve et al., 2011). Therefore, we set a buffer zone of 1,000 km around the minimum convex polygon composed of solitary desert locust samples to limit the sampling range of background points. This distance corresponds to the maximum distance between most of the outbreak areas and the recession areas in the desert locust occurrence record dataset (Meynard et al., 2017). The maximum number of background points used for sampling was 10,000. To reduce uncertainty, the bootstrap data segmentation method was repeated 10 times for modeling (Moraitis, Valavanis & Karakassis, 2019). The maximum number of iterations was set to 1,000 so that the model had enough time to reach convergence (Zhang et al., 2020).

The logistic format was selected for the output, which gives an estimate of the probability of species existence. The logistic output value is continuous and ranges from 0 to 1 (Phillips et al., 2006; Phillips & Dudík, 2008). We chose the threshold under the “maximum training sensitivity plus specificity” (MTSS) criterion recommended by MaxEnt and converted the continuous suitability score output by MaxEnt into binary suitability. The criterion uses training data to optimize the balance between specificity and sensitivity, so it is considered one of the best threshold selection methods (Liu, Newell & White, 2016; Shabani, Kumar & Ahmadi, 2018). Subsequently, the suitability results were divided into four categories: unsuitable (0-MTSS), low suitability (MTSS-0.4), moderate suitability (0.4–0.6), and high suitability (0.6–1) (Liao et al., 2020). Considering the availability of data and the limited impact of human activities and natural processes on the soil sand content and topography at coarse resolution (0.5° × 0.5°) in the short term. For future scenarios, we assume that the values of the terrain and soil variables in the future are consistent with those in the current dataset, and the remaining bioclimatic variables come from eight GCM projections in the CMIP6 model (Guo et al., 2019; Wang et al., 2019). The potential habitats of desert locusts in the 2050s and 2070s under each future scenario (SSP126, SSP245, SSP370, and SSP585) were simulated. The equal-weighted average of the eight GCM results was defined as the average predicted probability of future potential habitats, and the results were classified according to the above principles.

### Model evaluation

We used the AUC and true skill statistic (TSS) for model evaluation (Fielding & Bell, 1997; Allouche, Tsoar & Kadmon, 2006). AUC is a threshold-independent statistic that is used to differentiate presence from absence to evaluate model performance (Thuiller, Lavorel & Araujo, 2005). The AUC value is usually divided into five levels (Swets, 1988; Li et al., 2019; Wang et al., 2019): 0–0.6 (fail), 0.6–0.7 (poor), 0.7–0.8 (fair), 0.8–0.9 (good), and 0.9–1 (excellent). In the actual application process, it is usually necessary to determine an appropriate threshold value and convert the continuous prediction results into binary presence and absence results. At this time, the TSS is a simple and intuitive method to measure the performance of SDMs (Allouche, Tsoar & Kadmon, 2006; Shabani, Kumar & Ahmadi, 2018). The TSS depends on a threshold. We selected the threshold under the MTSS standard to calculate the TSS. The TSS ranges from −1 to 1, where one means that the observed value is the same as the predicted value, and a value of 0 or less indicates that the performance is not better than random (Allouche, Tsoar & Kadmon, 2006). AUC evaluation has the advantage of threshold independence and has been widely used, but this evaluation indicator is sometimes criticized because it equally weights omission and commission errors (Lobo, Jiménez-valverde & Real, 2008). Therefore, we used both AUC and TSS values to improve the performance of the model.

Due to the uncertainty of the future climate predicted by different GCMs, this uncertainty may affect the prediction accuracy of the desert locust habitat. Therefore, we introduced a majority voting approach to further reduce the uncertainty in the prediction results of desert locust habitats under different scenarios (SSP126, SSP245, SSP370, and SSP585) in the 2050s and 2070s (Li et al., 2019). First, we superimposed the prediction results of the desert locust habitat under the eight GCMs projections according to different periods and different scenarios. Then, the number of unanimous votes on the prediction results of desert locust habitats under different periods and different scenarios was counted. The higher the final consensus number, the lower the uncertainty of the prediction results, and vice versa.

## Results

### Model evaluations and contributions of the variables

By comparing the AUC values of the MaxEnt model under different regularization multipliers and feature combinations, we found that the model performs best when the regularization multiplier is one and the feature combination is LQHPT (Table S2). Therefore, the regularization multiplier was set to one, and the feature combination was set to LQHPT during the modeling process. We determined the AUC and TSS values from 10 replicate runs to check the model performance. The average AUC value and TSS value reached 0.908 ± 0.002 and 0.701, respectively, indicating that the model has excellent performance and outstanding prediction results (Swets, 1988; Allouche, Tsoar & Kadmon, 2006).

**Figure 2:**
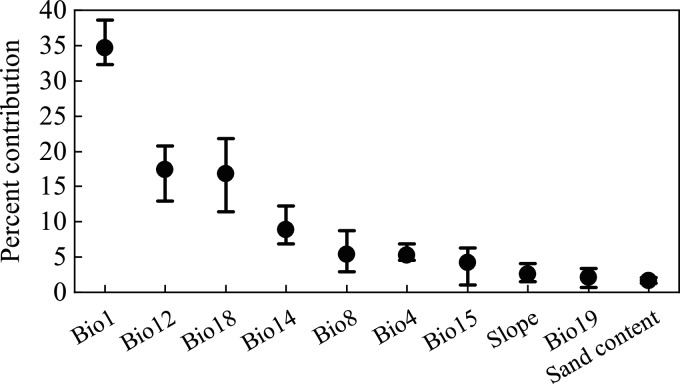
Percentage contribution of environmental variables in the MaxEnt model. The whiskers indicate the range of 10 repeated runs, and the black dots indicate the mean value.

Figure 2 shows the percentage contribution of variables after 10 replicate runs of MaxEnt. Among all the variables, the six highest-ranked were annual average temperature (Bio1, 34.8%), annual precipitation (Bio12, 17.5%), mean precipitation of the warmest quarter (Bio18, 16.9%), precipitation of the driest month (Bio14, 9.0%), mean temperature of wettest quarter (Bio8, 5.5%), and temperature seasonality (Bio4, 5.3%); the average cumulative contributions of these variables exceeded 89% (Fig. 2). The contributions of the other environmental variables were relatively small.

**Figure 3:**
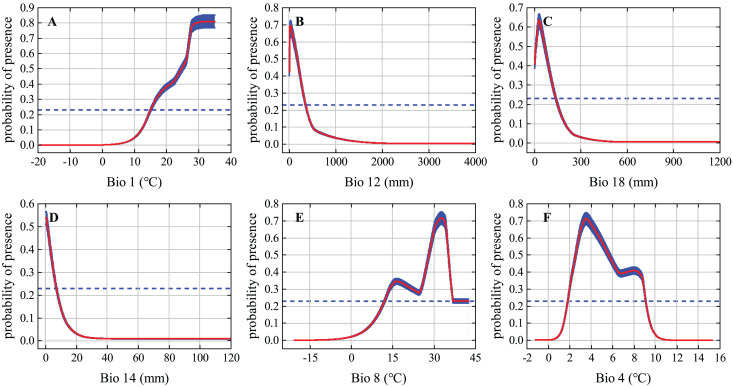
Response curves of the six most important environmental variables. The red curve and blue shading show the mean response of the 10 replicate Maxent runs and the mean +/− one standard deviation, respectively; the blue dashed line represents the maximum training sensitivity plus specificity (MTSS) threshold.

The response curves of the variables reflect how each environmental variable affects MaxEnt predictions. We generated individual response curves for the six most important environmental variables to determine the environmental preferences of the desert locust during the solitary period. The response curves of the environmental variables show obvious characteristics of change. The habitat suitability of desert locusts is positively correlated with Bio1 but is generally negatively correlated with Bio12, Bio18, and Bio14. Under the MTSS threshold, the suitable ranges of Bio1, Bio12, Bio18, Bio14, Bio8, and Bio4 were 15–35 °C, 0–350 mm, 0–135 mm, 0–7 mm, 12–42.5 °C, and 1.8–9.1 °C, respectively (Fig. 3). Areas within these environmental thresholds are more suitable for the survival of solitary desert locusts.

### Habitat suitability under current conditions

**Figure 4:**
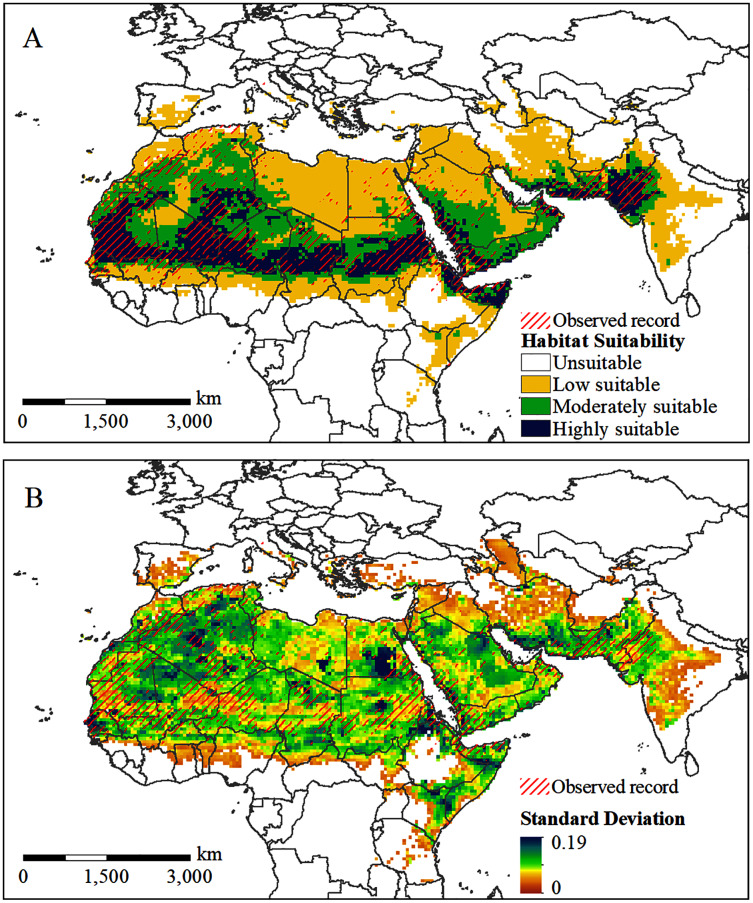
Predicted potential habitat of desert locust under current conditions. (A) The predicted probability of occurrence; (B) the standard deviation between the results of 10 repeated runs.

The potential distribution of the solitary desert locust under current conditions is shown in Fig. 4. The predicted range of potential habitats (moderately and highly suitable areas) is consistent with the observed occurrences of solitary desert locusts (Fig. 4A). The standard deviation between the results of multiple runs of the model is generally small, indicating that the model is robust (Fig. 4B). However, the model underestimated the probability of occurrence in very few areas, such as central Algeria, southern Libya, southern Egypt, and northern Saudi Arabia. Although lone desert locusts in the solitary stage were observed in these areas, they were assessed as areas with low suitability (Fig. 4A). Almost all the overestimated or underestimated areas are distributed in the transitional area between the areas with low and moderate suitability. This is also where the standard deviation between the results of multiple runs of the model is the largest (Fig. 4B).

Figure 4A shows that the potentially suitable areas for solitary desert locusts are concentrated spatially and cover a wide range, extending from West Africa to the Indian Peninsula. Highly suitable areas are mainly distributed in most of Mauritania, central and eastern Mali, southern Algeria, southern Niger, central Chad, central Sudan, northern Somalia, western Saudi Arabia, western Yemen, southern Iran, and the India-Pakistan border. On the African continent, a long and narrow area with moderate suitability is formed south of the highly suitable area, while the range of the moderately suitable area in the north is relatively wide, mainly concentrated in central Algeria, eastern Mauritania, western Mali, northern Niger, northern Chad, and northern Sudan. In addition, there are large areas of moderately suitable areas in southern Saudi Arabia, Yemen, Oman, and the India-Pakistan border. Areas with low suitability are mainly distributed on the periphery of moderately suitable areas, especially in Morocco, northern Algeria, Libya, Egypt, the northern Arabian Peninsula, central-eastern Iran, western India, and the border of Ethiopia, Somalia, and Kenya.

### Habitat suitability under future conditions

**Figure 5:**
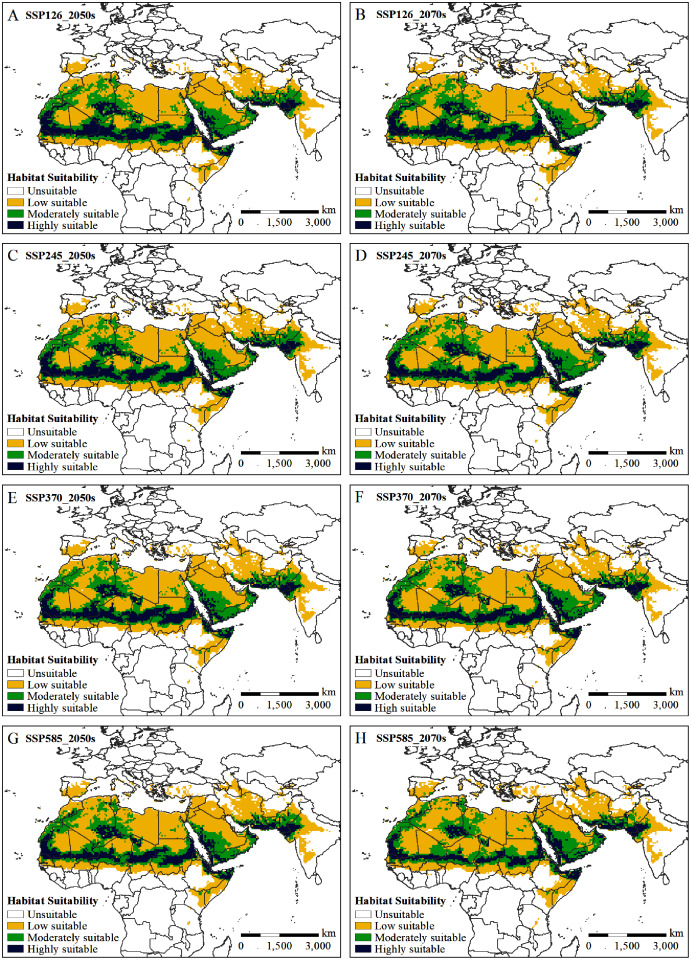
(A–H) Predicted potential habitat of the desert locust in two time periods (2050s and 2070s) under four climate scenarios (SSP126, SSP245, SSP370, and SSP585).

**Figure 6:**
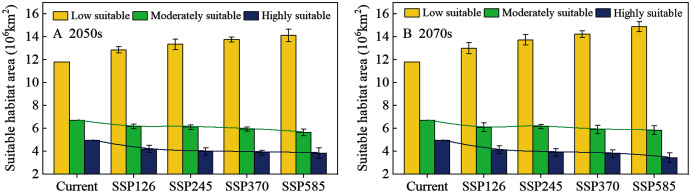
The area of suitable habitat for desert locusts under different scenarios in the (A) 2050s and (B) 2070s. The whiskers represent the standard deviation of the results of eight GCM projections.

The potential distribution of solitary desert locusts in the 2050s and 2070s under the SSP126, SSP245, SSP370, and SSP585 scenarios was predicted by the MaxEnt model. Fig. 5 shows the impact of different climate change scenarios on the potential distribution of desert locusts. Compared with the current conditions, under the most conservative scenario (SSP126), the moderately and highly suitable areas in the 2050s will be reduced by 0.55 × 10^6^ km^2^ and 0.76 × 10^6^ km^2^, respectively, while in the 2070s, they will be reduced by 0.62 × 10^6^ km^2^ and 0.84 × 10^6^ km^2^, respectively (Figs. 5A, 5B, 6). As the radiative forcing increases, this contraction becomes more prominent. Under the SSP585 scenario, the moderately and highly suitable areas will decrease by 1.07 × 10^6^ km^2^ and 1.13 × 10^6^ km^2^ in the 2050s, respectively, and the amount of contraction will reach 0.88 × 10^6^ km^2^ and 1.55 × 10^6^ km^2^ in the 2070s, respectively (Figs. 5G, 5H, 6). In all scenarios and periods, the low suitable areas show an expansion trend. The increased area is mainly obtained in two ways: the shift of the moderately suitable areas and the expansion of the areas with low suitable at the edge of the recession regions. In general, with the increase in radiative forcing and time, the potentially suitable areas (moderately and highly suitable areas) for solitary desert locusts will contract significantly.

**Figure 7:**
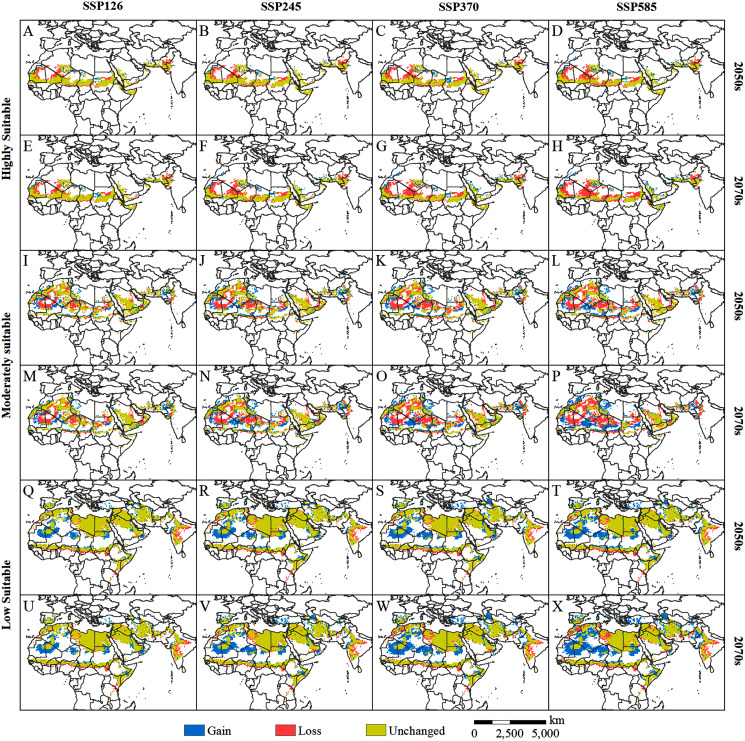
(A–X) Changes in the potential habitat of the desert locust relative to the current habitat under different climate scenarios in the 2050s and 2070s.

By comparing the current (Fig. 4) and predicted distributions of the suitable habitats for solitary desert locusts in the middle and late 21st century (Fig. 5), we analyzed the spatial changes in the potentially suitable habitats for this species under different climate scenarios (Fig. 7). In the 2050s, the highly suitable areas will decrease by 1.15 × 10^6^–1.70 × 10^6^ km^2^, and by the 2070s, the decrease in the area will reach 1.26 × 10^6^–2.28 × 10^6^ km^2^. The further contraction will occur in central Mauritania, northeastern Mali, southern Algeria, central Chad, central Sudan, and the India-Pakistan border. The areas of expansion for the highly suitable regions are mainly scattered in southern Morocco, southern western Sahara, eastern Algeria, northern Chad, western Sudan, western Saudi Arabia, southern Iran, and southern Pakistan, with areas of only 0.39 × 10^6^–0.57 × 10^6^ km^2^ in the 2050s and 0.42 × 10^6^–0.73 × 10^6^ km^2^ in the 2070s (Figs. 7A–7H, Table 2). The moderately suitable areas will decrease by 2.07 × 10^6^–3.03 × 10^6^ km^2^ and 2.20 × 10^6^–3.61 × 10^6^ km^2^ in the 2050s and 2070s, respectively. Losses in the moderately suitable areas will occur mainly in eastern Mauritania, northern Mali, central Algeria, northern Niger, northern Sudan, and western India. At the same time, climate change will cause the moderately suitable areas to increase by 1.52 × 10^6^–1.96 × 10^6^ km^2^ and 1.58 × 10^6^–2.73 × 10^6^ km^2^ in the 2050s and 2070s, respectively. These gains are derived mainly from the highly suitable areas (Figs. 7I–7P, Table 2). In the 2050s, the areas with low suitability will expand by 2.42×10^6^–3.81×10^6^ km^2^, and by the 2070s, the expanded areas will reach 2.57 × 10^6^–5.25 × 10^6^ km^2^. The expansion areas are mainly derived from moderately and highly suitable areas. The areas of contraction were small and were mainly scattered around the periphery of the areas with low suitability (Figs. 7Q–7X, Table 2). In general, for the solitary desert locust, habitat loss will become more prominent with increasing radiative forcing and time, which will lead to a significant contraction of its suitable habitat area by the end of the 21st century.

**Table 2:** Changes in the potential habitat area of desert locusts relative to the current area under different climate scenarios.

Scenarios	Time	Low suitable area (×10^6^ km^2^)			Moderately suitable area (×10^6^ km^2^)			Highly suitable area (×10^6^ km^2^)		
		Gain	Loss	Unchanged	Gain	Loss	Unchanged	Gain	Loss	Unchanged
SSP126	2050s	2.42	1.34	10.48	1.52	2.07	4.68	0.39	1.15	3.84
	2070s	2.57	1.35	10.48	1.58	2.20	4.55	0.42	1.26	3.74
SSP245	2050s	2.99	1.44	10.38	1.79	2.39	4.36	0.41	1.39	3.61
	2070s	3.56	1.61	10.22	2.12	2.67	4.07	0.58	1.64	3.36
SSP370	2050s	3.39	1.42	10.40	1.83	2.61	4.14	0.45	1.53	3.46
	2070s	4.31	1.87	9.96	2.27	3.10	3.65	0.68	1.87	3.13
SSP585	2050s	3.81	1.48	10.35	1.96	3.03	3.72	0.57	1.70	3.29
	2070s	5.25	2.14	9.69	2.73	3.61	3.13	0.73	2.28	2.70

## Discussion

### Sampling range of background points and model performance

Previous studies have shown that model performance will have obvious differences in response to different background point sampling ranges (Anderson & Raza, 2010; Barve et al., 2011; Merow, Smith & Silander, 2013). We ran the MaxEnt model with the same parameter conditions for three background points sampling ranges: (1) the global scale, (2) Asia, Europe, and Africa, and (3) a 1,000 km buffer zone surrounding the minimum convex polygon formed by species occurrence records. The AUC values of the three models were 0.966 ± 0.001, 0.940 ± 0.001, and 0.908 ± 0.002. With the expansion of the study range, the AUC value of the MaxEnt model gradually increased. However, the accuracy of the model may be overestimated as the study range expands. For the same model, different ranges of background point data are used for evaluation, and the accuracy of the model will be improved as the sampling range of the background test data expands. Therefore, when constructing a model, the background range should only include the locations that the species may reach, rather than using a very large range (Anderson & Raza, 2010; Merow, Smith & Silander, 2013). We ultimately chose the third range as the background points sampling range. This distance corresponds to the maximum distance between most outbreak areas and recession areas in the desert locust occurrence record database (Meynard et al., 2017). Therefore, the model we built based on this range has strong rationality.

MaxEnt’s simulation of the potential habitat for solitary desert locusts under current conditions is very similar to the observation records. However, the model underestimates the occurrence probability of desert locusts in local areas, such as central Algeria, southern Libya, southern Egypt, and northern Saudi Arabia (Fig. 4A). This may be due to some uncertainties in the desert locust occurrence records or the very low density of solitary individuals, which may cause them to be missed during investigations (Renier et al., 2015; Meynard et al., 2017). In addition, desert locusts have strong environmental adaptability and can survive in extreme weather conditions for short periods (Cressman & Stefanski, 2016). This uncertainty may explain the partial underestimation by Maxent. However, our models still resulted in a good map, consistent with the known distribution of the species.

### Consistency of environmental variables and desert locust ecology

All the different phases of the desert locust’s life cycle require ideal meteorological conditions, and these conditions allow the insect to develop from the solitary phase to the gregarious phase and cause widespread harm (Cressman & Stefanski, 2016). Rainfall and temperature are some of the most important environmental conditions affecting the survival of desert locusts. In fact, several of the environmental variables that contributed most to our modeling of solitary desert locust distribution were characterized by precipitation and temperature (Fig. 3). Rainfall is of great significance to the reproduction and migration of desert locusts. Female desert locusts usually prefer to lay their eggs in warm, sandy, and moist soil. Appropriate rainfall is conducive to the hatching, development, and reproduction of desert locusts. It is generally believed that more than 25 mm of rainfall for two consecutive months is sufficient for locusts to reproduce and develop (Symmons & Cressman, 2001; Tratalos et al., 2010; Cressman & Stefanski, 2016). The model results show that the most suitable ranges of annual precipitation (Bio12), mean precipitation of the warmest quarter (Bio18), and precipitation of the driest month (Bio14) for solitary desert locusts are 0–350 mm, 0–135 mm, and 0–7 mm, respectively (Fig. 3). Previous studies have suggested that during the recession period, desert locusts are usually confined to the semiarid and arid desert regions of Africa, the Middle East, and Southwest Asia, where the annual precipitation is usually less than 200 mm (Uvarov, 1997; Cressman & Stefanski, 2016). However, in areas suitable for solitary desert locusts such as the Red Sea coast, and the India-Pakistan border, the annual precipitation reaches 200–500 mm (Wang et al., 2020). Although these areas are relatively small, the MaxEnt model still recognized this detail. Therefore, our results are consistent with existing conclusions and experiences.

Temperature is a function of the development speed of eggs and nymphs, and it is also the basic condition for adults to take off and flight (Symmons & Cressman, 2001; Cressman & Stefanski, 2016). Usually, within the optimal culture range, as the temperature increases, the time for the development of desert locust egg embryos gradually shortens, and the eggs develop faster under high-temperature conditions (Huque & Jaleel, 1970). Eggs are less likely to hatch when the soil temperature is below 15 °C, while embryos may die when the soil temperature is above 35 °C (Cressman & Stefanski, 2016). The migration of solitary desert locusts occurs at night when the temperature is higher than 20–22 °C. Continuous flight requires warm temperatures, and continuous flight at temperatures below 20 °C is rare (Symmons & Cressman, 2001; Cressman & Stefanski, 2016). However, the ecological requirements of swarms are much lower than those of solitary locusts, so gregarious desert locusts are more resistant to high temperature and drought and can withstand temperatures of 40 °C and above (Uvarov, 1997). Our results show that the optimal annual mean temperature (Bio1) and mean temperature of the wettest quarter (Bio8) for solitary desert locusts are 15–35 °C and 12–42.5 °C, respectively, which is consistent with the findings of existing studies. In summary, our findings concerning the ecology of the solitary desert locust are reliable.

### Changes in geographical distribution under climate change scenarios

The prediction based on multiple GCMs of the CMIP6 model shows that with an increase in radiative forcing and time, the suitable habitat of the solitary desert locust will contract significantly, especially in the 2070s under the SSP585 scenario. This trend of change is similar to the previous research conclusions of Meynard et al. (2017) and Saha, Rahman & Alam (2021). However, our research is slightly different from that of Saha, Rahman & Alam (2021) in terms of the distribution and range of changes in the highly suitable areas of the desert locusts. This may be related to the differences in species occurrence data and environmental variables used in the two studies. Climate change and extreme climatic conditions are the main driving factors for changes in desert locust habitats (Meynard et al., 2017; Gómez et al., 2020; Kimathi et al., 2020; Salih et al., 2020). The risk of future climate change in Africa is very high, and the ability to cope with future climate change is very tenuous (IPCC, 2014). Studies have shown that in the 21st century, climate change may cause the temperature in Africa to rise by 2 to 6 °C, with the greatest increase occurring in the Sahara Desert and the semiarid tropical fringe of central Africa (Hulme et al., 2001). Under the SSP126, SSP245, and SSP585 scenarios, the temperature of the Sahara in 2030–2059 (2070–2099) will increase by 1.6 (1.6) °C, 1.8 (2.9) °C, and 2.2 (5.3) °C, respectively, compared with that in 1981–2010. In the same scenarios and periods, the projected temperature rise in West Africa is 1.2 (1.3) °C, 1.4 (2.4) °C, and 1.7 (4.2) °C, respectively (Almazroui et al., 2020). In addition, the risk of drought in various parts of Africa is expected to increase in the future, and arid regions are expected to expand further, while the risk of drought is higher in North and Central African countries (Russo et al., 2016; Ahmadalipour et al., 2019). The abovementioned areas of environmental deterioration correspond to areas with reduced potential habitats of solitary desert locusts (eastern Mauritania, northern Mali, southern Algeria, central Chad, northern Niger, and northern Sudan, Fig. 7). Continuous high temperatures and droughts have destroyed the habitats of desert locusts, such as those used for breeding and foraging, causing the moderately and highly suitable areas to shrink continuously.

Although the potentially suitable areas for desert locusts are contracting, due to the following considerations, the future impacts of desert locusts on agricultural production and food security should not be underestimated. First, traditional summer (such as southern Mauritania, central Mali, western Niger, central Chad, central Sudan, and the India-Pakistan border) and winter (such as western Algeria, northwestern Mauritania, the Red Sea coast of Sudan and Saudi Arabia, the Somali Peninsula, southern Iran, and southern Pakistan) breeding areas are still projected to be conducive to the reproduction of desert locusts (Fig. 5). This means that the breeding source of desert locusts still exists on a large scale. Second, some areas highly suitable for solitary desert locusts are expected to expand in parts of southern Morocco, southern Western Sahara, eastern Algeria, northern Chad, western Sudan, western Saudi Arabia, southern Iran, and southern Pakistan (Fig. 7). Given the high tolerance of desert locusts to environmental variation, these small highly suitable areas may still become a source of swarm outbreaks under suitable climatic conditions (such as extreme precipitation). Third, extreme precipitation events can create favorable conditions for accelerated reproduction and gathering of desert locusts (Ceccato et al., 2007; Salih et al., 2020; Meynard et al., 2020). Research has shown that extreme precipitation events will increase in parts of Africa in the 21st century (Donat et al., 2016; Tegegne, Melesse & Alamirew, 2021). The frequent occurrence of extreme precipitation events will increase the risk of desert locust plagues. Fourth, population growth will intensify agricultural expansion, reduce the living space of desert locusts, and increase agricultural exposure to locust plagues. Research has shown that by the end of this century, the population of Africa is expected to increase to between 3.1 and 5.7 billion, with a probability of 95% (Gerland et al., 2014). This unprecedented population increase will pose serious challenges to food security. In addition, due to the long-term sociopolitical instability and continuous armed conflicts in some countries in the Middle East and East Africa, investment in early monitoring and early warning and control of desert locusts is very limited, which worsens the challenge of preventing and controlling desert locust disasters (Salih et al., 2020; Meynard et al., 2020). Based on the above concerns, we believe that even though the potential habitat of the solitary desert locust is contracting, its threat to agricultural development and food security is still strong.

### Uncertainties and prospects

**Figure 8:**
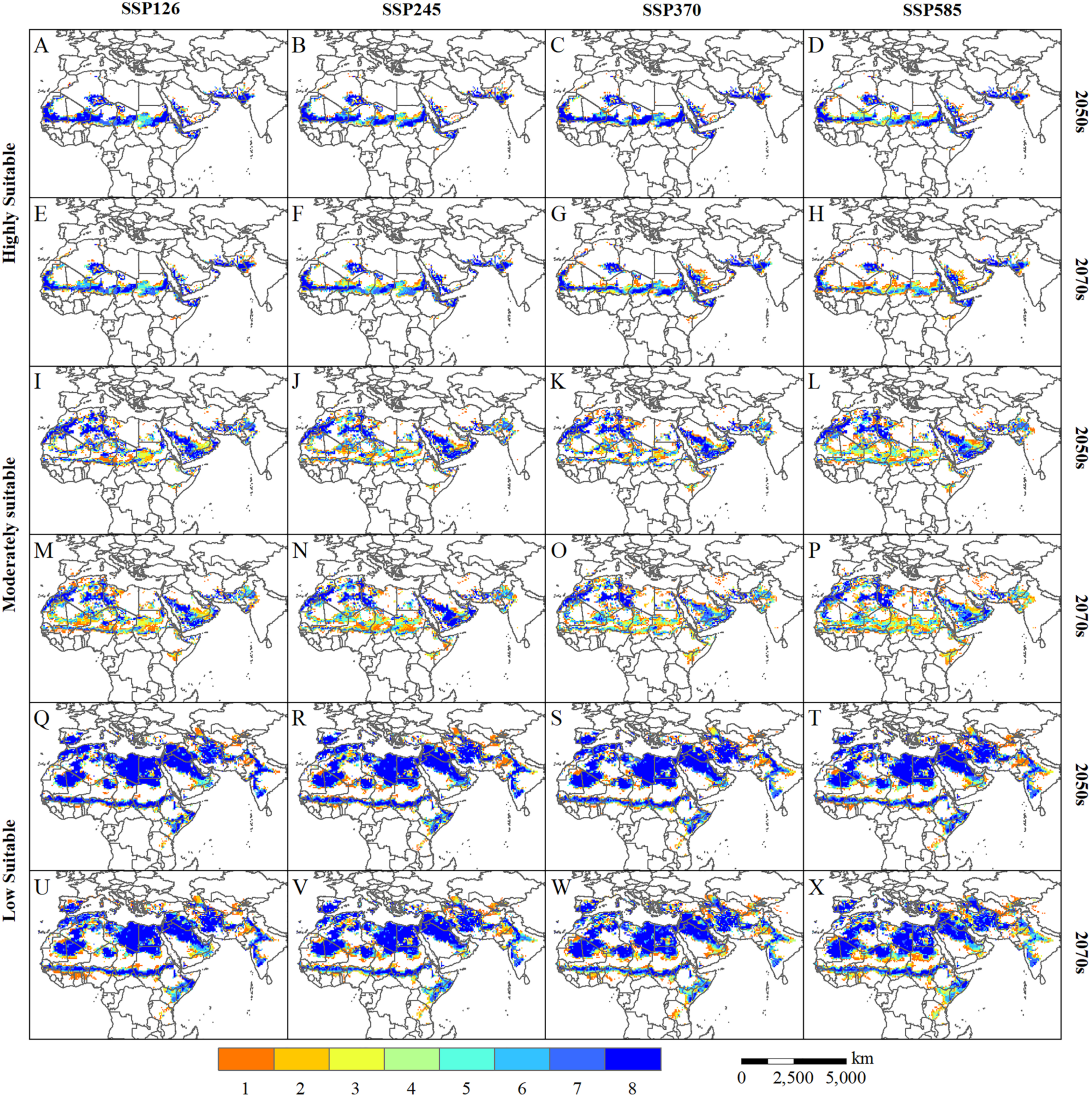
(A–X) Voting consensus map of potentially suitable areas for desert locusts in the 2050s and 2070s based on eight GCMs climate data. The value (1–8) represents the number of voting consensus. The larger the value, the higher the consistency of the simulation results. For example, a pixel value of eight indicates that all simulation results agree that the pixel is a potential habitat for desert locusts.

The uncertainty of the future climate affects the projection accuracy of the desert locust habitat, and the prediction results may confuse policymakers. Therefore, we used the majority voting approach to further reduce the uncertainty, to better provide a reference for the monitoring, early warning, and control of desert locusts. Figure 8 shows the consensus map of the potentially suitable areas for desert locusts in the 2050s and 2070s under the projection of eight GCMs. The higher the consensus number, the lower the uncertainty, and vice versa. On the whole, the uncertainty of the highly suitable areas and the low suitable areas is lower, while the uncertainty of the moderately suitable areas is higher. Among them, the uncertainty in central Sudan, central Chad, and the Arabian Peninsula in the highly suitable areas are slightly higher, especially in the 2070s under the SSP585 scenario (Fig. 8H). In the low suitable areas, Kenya, Tanzania, northern Afghanistan, and the India-Pakistan border have higher uncertainties (Figs. 8Q–8X). Among the moderately suitable areas, the areas with higher uncertainty mainly appear in the southern Sahel, the eastern part of Saudi Arabia, and the India-Pakistan border (Figs. 8I–8P). In general, the uncertainty of the desert locust’s potential habitat will increase with time and radiative forcing (Fig. 8A *vs*. Fig. 8H; Fig. 8I *vs*. Fig. 8P; Fig. 8Q *vs*. Fig. 8X).

In addition, the MaxEnt model we used to assume unlimited dispersal when predicting species distributions. However, in addition to climate and soil factors, the potential distribution of desert locusts may be restricted by biological factors (such as natural enemies and vegetation), human activities (such as land use, anthropogenic prevention and control), and other factors. The result, which does not account for dispersal limitation, is a prediction of only the potential suitable area, not the habitat that the species can actually occupy in the future. Therefore, our model may have overestimated the potential distribution range of the solitary desert locust in the future.

Desert locusts, whether solitary or gregarious, have a strong migratory ability (Symmons & Cressman, 2001; Cressman & Stefanski, 2016), which causes great uncertainty in the accurate prediction of their habitat. Therefore, future research should focus on accurately grasping the migratory laws of desert locusts. Combining climate change and human activity factors, diffusion constraints were set according to the habits of desert locusts in different stages, using a variety of scenarios to accurately predict the actual habitats occupied by this species.

## Conclusions

Based on MaxEnt, we successfully established a species distribution model that comprehensively considers environmental variables such as climate, soil, and terrain. This model was used to study the impact of climate change on the habitat of desert locusts. The potential habitats of solitary desert locusts under four climate change scenarios in the CMIP6 model were predicted in the present, 2050s and 2070s. Our results indicate that climate change will have an impact on the distribution of the potential habitat for solitary desert locusts in the 2050s and 2070s. With the increase in radiative forcing and time, the areas moderately and highly suitable for desert locusts will continue to contract, especially in the 2070s under the SSP585 scenario. Although the potentially suitable area for desert locusts is contracting, the future threat to agricultural production and food security from desert locust plagues should not be underestimated due to the maintenance of breeding areas, frequent occurrence of extreme weather events, pressure from population growth, and turbulent sociopolitical environment. These results have considerable value as a reference for desert locust monitoring, early warning, and control.

## Supplemental Information

10.7717/peerj.12311/supp-1 Supplemental Information 1 Raw occurrence data. Occurrence data after cleaning and the final sample data used for modeling.

10.7717/peerj.12311/supp-2 Supplemental Information 2 Initially selected environment variables.

10.7717/peerj.12311/supp-3 Supplemental Information 3 Model performance under different regularization multiplier and feature combination.

## Additional Information and Declarations

### Data Availability

The following information was supplied regarding data availability: The raw data is available in the Supplementary File.
