# Supplementary material for: The potential habitat of desert locusts is contracting: predictions under climate change scenarios
Source: PeerJ. 2021 Oct 26;9:e12311. doi: 10.7717/peerj.12311 (PMC8555501; doi:10.7717/peerj.12311)
Supplement: Supplemental Information 2 [file peerj-09-12311-s002.docx]

**Table S1**

Initially selected environment variables.

| Variable | Describe |
| --- | --- |
| Bio1 | Annual mean temperature (℃) |
| Bio2 | Monthly diurnal range (℃) |
| Bio3 | Isothermally (%) |
| Bio4 | Temperature seasonality (standard deviation ×100) |
| Bio5 | Max temperature of the warmest month (℃) |
| Bio6 | Min temperature of coldest month (°C) |
| Bio7 | Annual range of temperature (℃) |
| Bio8 | Mean temperature of wettest quarter (°c) |
| Bio9 | Mean temperature of the driest quarter (℃) |
| Bio10 | Mean temperature of warmest quarter (°c) |
| Bio11 | Mean temperature of coldest quarter (°c) |
| Bio12 | Annual Precipitation (mm) |
| Bio13 | Precipitation of wettest month (mm) |
| Bio14 | Precipitation of the driest month (mm) |
| Bio15 | Precipitation seasonality (Coefficient of Variation) |
| Bio16 | Precipitation of wettest quarter (mm) |
| Bio17 | Precipitation of driest quarter (mm) |
| Bio18 | Mean precipitation of the warmest quarter (mm) |
| Bio19 | Mean precipitation of the coldest quarter (mm) |
| Alt | Altitude (m) |
| Slope | Slope (°) |
| Aspect | Aspect (°) |
| T-SAND | Topsoil Sand Fraction (% wt.) |
