# Supplementary material for: The potential habitat of desert locusts is contracting: predictions under climate change scenarios
Source: PeerJ. 2021 Oct 26;9:e12311. doi: 10.7717/peerj.12311 (PMC8555501; doi:10.7717/peerj.12311)
Supplement: Supplemental Information 3 [file peerj-09-12311-s003.docx]

**Table S2**

Model performance under different regularization multiplier and feature combination.

| **Regularization multiplier** | **Feature combination** | **Train AUC** | **Test AUC** |
| --- | --- | --- | --- |
| 0.5 | L | 0.870 | 0.877 |
| 0.5 | LQ | 0.876 | 0.886 |
| 0.5 | H | 0.905 | 0.896 |
| 0.5 | LQH | 0.904 | 0.901 |
| 0.5 | LQHP | 0.904 | 0.897 |
| 0.5 | LQHPT | 0.906 | 0.900 |
| 1 | L | 0.867 | 0.874 |
| 1 | LQ | 0.875 | 0.880 |
| 1 | H | 0.900 | 0.904 |
| 1 | LQH | 0.901 | 0.902 |
| 1 | LQHP | 0.904 | 0.903 |
| **1** | **LQHPT** | **0.908** | **0.904** |
| 1.5 | L | 0.871 | 0.871 |
| 1.5 | LQ | 0.874 | 0.879 |
| 1.5 | H | 0.900 | 0.900 |
| 1.5 | LQH | 0.899 | 0.898 |
| 1.5 | LQHP | 0.901 | 0.895 |
| 1.5 | LQHPT | 0.903 | 0.896 |
| 2 | L | 0.870 | 0.872 |
| 2 | LQ | 0.873 | 0.885 |
| 2 | H | 0.901 | 0.890 |
| 2 | LQH | 0.899 | 0.892 |
| 2 | LQHP | 0.899 | 0.899 |
| 2 | LQHPT | 0.903 | 0.892 |
| 2.5 | L | 0.870 | 0.874 |
| 2.5 | LQ | 0.875 | 0.878 |
| 2.5 | H | 0.898 | 0.898 |
| 2.5 | LQH | 0.898 | 0.898 |
| 2.5 | LQHP | 0.898 | 0.899 |
| 2.5 | LQHPT | 0.901 | 0.898 |
| 3 | L | 0.870 | 0.875 |
| 3 | LQ | 0.874 | 0.878 |
| 3 | H | 0.897 | 0.897 |
| 3 | LQH | 0.897 | 0.897 |
| 3 | LQHP | 0.897 | 0.898 |
| 3 | LQHPT | 0.899 | 0.897 |
| 3.5 | L | 0.870 | 0.875 |
| 3.5 | LQ | 0.874 | 0.878 |
| 3.5 | H | 0.896 | 0.896 |
| 3.5 | LQH | 0.895 | 0.896 |
| 3.5 | LQHP | 0.896 | 0.897 |
| 3.5 | LQHPT | 0.898 | 0.897 |
| 4 | L | 0.870 | 0.875 |
| 4 | LQ | 0.874 | 0.878 |
| 4 | H | 0.895 | 0.895 |
| 4 | LQH | 0.895 | 0.896 |
| 4 | LQHP | 0.895 | 0.897 |
| 4 | LQHPT | 0.897 | 0.896 |
| 4.5 | L | 0.870 | 0.875 |
| 4.5 | LQ | 0.874 | 0.878 |
| 4.5 | H | 0.895 | 0.895 |
| 4.5 | LQH | 0.895 | 0.896 |
| 4.5 | LQHP | 0.895 | 0.897 |
| 4.5 | LQHPT | 0.897 | 0.896 |
| 5 | L | 0.870 | 0.875 |
| 5 | LQ | 0.873 | 0.878 |
| 5 | H | 0.892 | 0.894 |
| 5 | LQH | 0.892 | 0.894 |
| 5 | LQHP | 0.893 | 0.895 |
| 5 | LQHPT | 0.896 | 0.895 |
| 5.5 | L | 0.870 | 0.875 |
| 5.5 | LQ | 0.873 | 0.877 |
| 5.5 | H | 0.891 | 0.893 |
| 5.5 | LQH | 0.891 | 0.893 |
| 5.5 | LQHP | 0.892 | 0.894 |
| 5.5 | LQHPT | 0.895 | 0.894 |
| 6 | L | 0.869 | 0.875 |
| 6 | LQ | 0.872 | 0.877 |
| 6 | H | 0.890 | 0.892 |
| 6 | LQH | 0.890 | 0.892 |
| 6 | LQHP | 0.891 | 0.893 |
| 6 | LQHPT | 0.894 | 0.893 |
| 6.5 | L | 0.869 | 0.875 |
| 6.5 | LQ | 0.871 | 0.876 |
| 6.5 | H | 0.889 | 0.891 |
| 6.5 | LQH | 0.889 | 0.892 |
| 6.5 | LQHP | 0.890 | 0.892 |
| 6.5 | LQHPT | 0.893 | 0.893 |
| 7 | L | 0.869 | 0.875 |
| 7 | LQ | 0.871 | 0.876 |
| 7 | H | 0.889 | 0.890 |
| 7 | LQH | 0.889 | 0.891 |
| 7 | LQHP | 0.889 | 0.891 |
| 7 | LQHPT | 0.892 | 0.892 |
| 7.5 | L | 0.869 | 0.875 |
| 7.5 | LQ | 0.870 | 0.875 |
| 7.5 | H | 0.888 | 0.890 |
| 7.5 | LQH | 0.888 | 0.890 |
| 7.5 | LQHP | 0.888 | 0.890 |
| 7.5 | LQHPT | 0.891 | 0.891 |
| 8 | L | 0.869 | 0.875 |
| 8 | LQ | 0.870 | 0.875 |
| 8 | H | 0.887 | 0.889 |
| 8 | LQH | 0.887 | 0.889 |
| 8 | LQHP | 0.887 | 0.889 |
| 8 | LQHPT | 0.890 | 0.891 |
| 8.5 | L | 0.869 | 0.875 |
| 8.5 | LQ | 0.870 | 0.875 |
| 8.5 | H | 0.886 | 0.889 |
| 8.5 | LQH | 0.886 | 0.888 |
| 8.5 | LQHP | 0.886 | 0.888 |
| 8.5 | LQHPT | 0.889 | 0.890 |
| 9 | L | 0.869 | 0.875 |
| 9 | LQ | 0.870 | 0.874 |
| 9 | H | 0.885 | 0.888 |
| 9 | LQH | 0.885 | 0.887 |
| 9 | LQHP | 0.885 | 0.887 |
| 9 | LQHPT | 0.888 | 0.889 |
| 9.5 | L | 0.869 | 0.875 |
| 9.5 | LQ | 0.869 | 0.874 |
| 9.5 | H | 0.884 | 0.887 |
| 9.5 | LQH | 0.884 | 0.886 |
| 9.5 | LQHP | 0.884 | 0.886 |
| 9.5 | LQHPT | 0.887 | 0.889 |
| 10 | L | 0.869 | 0.875 |
| 10 | LQ | 0.869 | 0.874 |
| 10 | H | 0.883 | 0.886 |
| 10 | LQH | 0.883 | 0.885 |
| 10 | LQHP | 0.883 | 0.885 |
| 10 | LQHPT | 0.886 | 0.888 |

Note: L = linear, Q = quadratic, H = hinge, P = product, and T = threshold.
